# Tumefactive Fat Necrosis with Multinucleate Giant Cell Reaction Mimicking Recurrent Renal Cell Carcinoma following Percutaneous Renal Cryoablation

**DOI:** 10.1155/2019/1678193

**Published:** 2019-02-26

**Authors:** Amanda E. Kahn, Kevin J. Wu, David D. Thiel

**Affiliations:** ^1^Department of Urology, Mayo Clinic, Jacksonville, FL, USA; ^2^Department of Pathology, Mayo Clinic, Jacksonville, FL, USA

## Abstract

Multinucleate Giant Cell (GC) reaction is a biological response that occurs secondary to infection, an implanted foreign body, tissue injury, or inflammation. In rare instances GC reactions have been reported following tissue ablation. Multinucleate GC reactions and tumefactive fat necrosis both have the ability to mimic cancer recurrence or metastasis and can appear as enhancing masses. We discuss a case of a surgically resected retroperitoneal perinephric mass thought to be recurrent renal cell carcinoma (RCC) that was pathologically confirmed as tumefactive fat necrosis with multinucleate GC reaction 2 years following percutaneous cryoablation of a small renal mass.

## 1. Introduction

Multinucleate Giant Cell (GC) reaction is typically a biological response to infection, tissue injury, or inflammation and can be noted following implantation of biomaterials or medical devices [1]. The GC reaction occurs when the immune system is activated causing merging of histiocytes, monocytes, and macrophages to become multinucleate cells that surround the foreign material or inflamed tissue [1–3]. Although multinucleate GC reactions are typically secondary to tissue damage and immune system activation, some GC reactions have been observed following ablation for the treatment of renal and liver lesions [4, 5]. We report our observation of a single patient that developed a delayed onset perinephric mass following percutaneous cryoablation that was pathologically noted to be multinucleate GC reaction with tumefactive fat necrosis following resection.

## 2. Case Presentation

**Figure 1:**
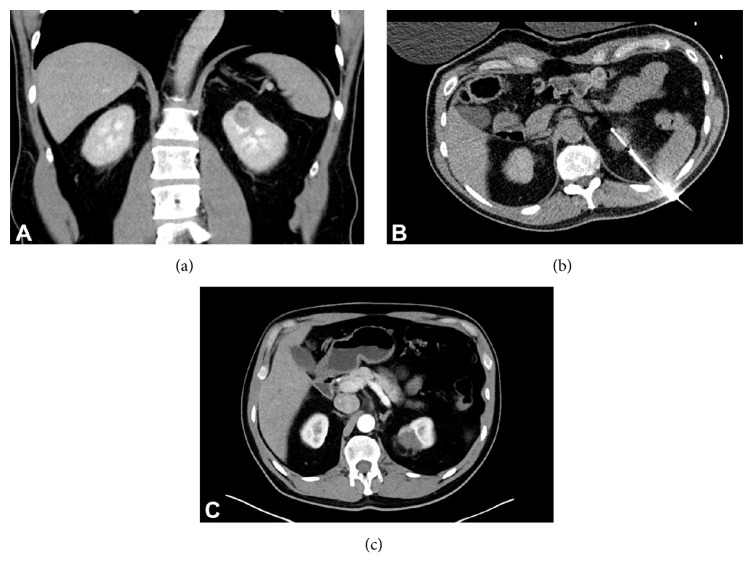
Patient imaging. (a) Contrast enhanced CT scan prior to ablation displaying 2-centimeter left renal mass in the upper pole. (b) An intra-therapy image of CT-guided left renal cryoablation probe displaying the tract of needle. (c) Contrast enhanced CT 1 year following cryoablation of left renal mass demonstrating absence of mass enhancement.

**Figure 2:**
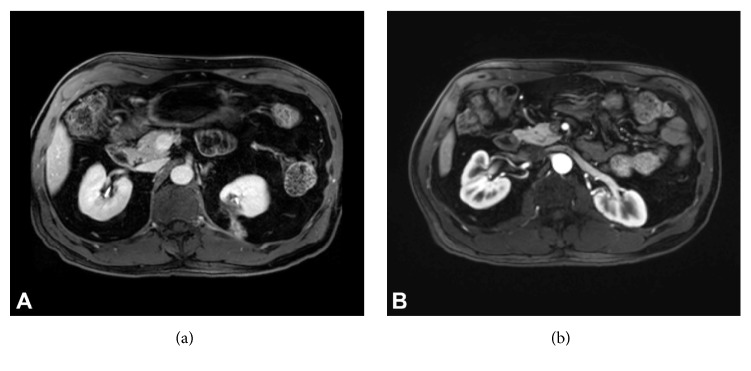
Patient imaging following cryoablation. (a) Contrasted MRI 2 years following cryoablation of left renal mass demonstrating new lesion development described as an ill-defined 2-centimeter focus of soft tissue highly suspicious for disease recurrence. The lesion appears to follow the cryoablation tract path. (b) Contrasted MRI 2-year status-post-partial nephrectomy of left renal lesion with full excision of perinephric fat displaying stable changes of bilateral partial nephrectomy without evidence of local recurrence.

**Figure 3:**
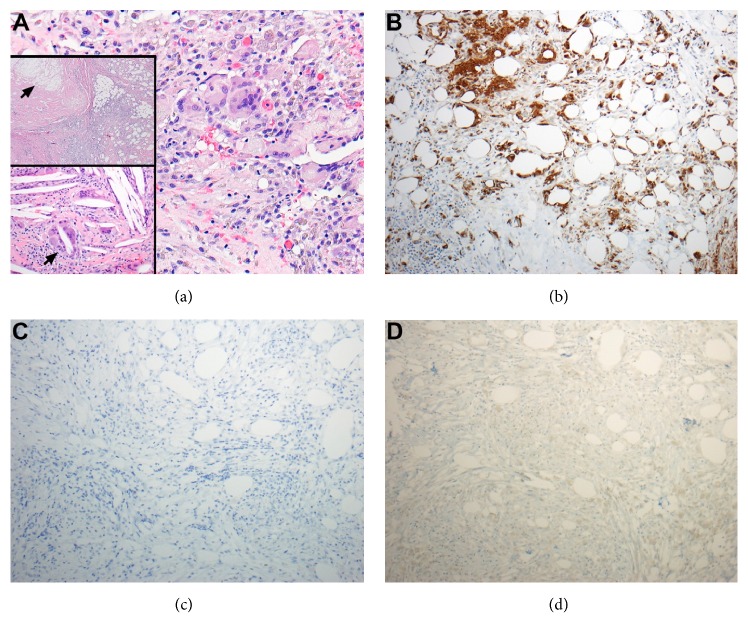
Pathology of lesion following partial nephrectomy. (a) Pathology of lesion showing area with prominent hemosiderin laden macrophages and multinucleate giant cells (H/E, 20X). Lower inset demonstrating foci of giant cells associated with cholesterol clefts (arrow) (H/E, 20X). Upper inset shows necrotic fat (arrow) with surrounding inflammatory reaction (H/E, 4X). (b) CD68 (KP1) immunohistochemistry (10X). CD68 is highly expressed by monocytes, macrophages, and histiocytes and is positive in numerous histiocytes and macrophages in adipose tissue. (c) Immunohistochemistry demonstrating a negative study for cytokeratin AE1/AE3 confirming no recurrence of renal cell carcinoma (10X). (d) Immunohistochemistry demonstrating a negative study for PAX-8 confirming no recurrence of renal cell carcinoma (10X).

A 60-year-old male had incidentally discovered bilateral renal masses identified on computed tomography (CT) imaging. Robotic assisted partial nephrectomy of the right renal lesion was completed on a 4-centimeter Furman Grade 2, pT1a clear cell RCC with negative surgical margins. Due to the posterior location of the 2-centimeter mass on the left kidney (Figure 1(a)), the patient opted for percutaneous cryoablation of the mass 4 months following the right robotic partial nephrectomy (Figure 1(b)). Follow-up cross-sectional imaging revealed no evidence of cancer recurrence in either kidney over a 2-year period (Figure 1(c)). A new contrast-enhancing lesion was noted in the perinephric fat of the left kidney 2 years following therapy (Figure 2(a)). Magnetic resonance imaging (MRI) scans also demonstrated a 2-centimeter enhancing and restricting ill-defined soft tissue focus in the left kidney region where previous cryoablation was performed. Biopsy was foregone due to the new rapid development, consistency, and location of the lesion. The patient underwent open subcostal partial nephrectomy with complete perinephric fat removal (Figure 2(b)). Surgical pathology revealed the resected renal mass to contain organized tumefactive fat necrosis with multinucleate GC reaction (Figure 3(a)) without evidence of clear cell RCC recurrence (Figures 3(c) and 3(d)). Immunohistochemistry studies (Figures 3(a) and 3(b)) demonstrated an inflammatory reaction characterized by abundant histiocytes and multinucleate giant cells that were positive for CD68 (KP1). Cytokeratin AE1/AE3 and PAX-8 immunohistochemistry were both negative (Figures 3(c) and 3(d)). The patient is 2 years removed from surgery without cancer or disease recurrence.

## 3. Discussion

Cryoablation is a favorable nephron-sparing treatment for certain renal lesions secondary to its ability to treat small, complex lesions in a minimally invasive fashion [4]. Our report describes a case of tumefactive fat necrosis with multinucleate GC reaction presented as a distinct mass in the perinephric tissue concerning for cancer recurrence. Our finding of tumefactive fat necrosis following cryoablation of a renal mass has been similarly reported by Durack et al. [6]. They describe 2 patients who presented with RCC, underwent cryoablation, and developed what looked like RCC recurrence. The patients underwent biopsy which confirmed both lesions as benign soft-tissue nodules with fat necrosis, fibrosis, inflammation, and histiocytes present [6]. The case demonstrates the benefit of biopsy, when possible, to avoid unnecessary excision of benign renal masses. This case study differs from our findings due to its lack of GC reaction but similarly reports patients who developed a delayed onset of fat necrosis following cryoablation.

The cellular disturbances that occur during cryoablative therapy can contribute to the development of a multinucleate GC reaction due to tissue damage, necrosis, and creation of cellular debris which stimulates an immune response [1]. A GC reaction in renal tissue has previously been observed by Johnson et al. who identified a patient that presented with RCC, underwent radiofrequency ablation, and developed a firm cortex at the site of ablation. A nephrectomy was performed 11 months following treatment secondary to the atrophic nature of the kidney revealing a granulomatous, foreign body GC reaction not detected on CT [4].

Also notable in our case was the presence of abundant fat necrosis. A study by Lokken et al. found that necrotic fat and inflammatory nodules have the ability to mimic tumor seeding or recurrence following cryoablation with delayed presentation in 2.7% of cases [7]. These nodules typically present in the tract of the cryoprobe and can display as an enhancing lesion on contrast-enhanced CT or MRI [7]. The unique combination of both multinucleate GC reaction and tumefactive fat necrosis may have contributed to the delayed presentation on imaging in our case allowing the reaction to mimic RCC recurrence in the cryoablative tract.

The histological appearance of our patient's radiographic abnormality following partial nephrectomy and perinephric fat excision is characterized by extensive tumefactive fat necrosis and multinucleate GC reaction at the site of previous ablative therapy (Figure 3(a)). As seen in Figure 3(b), CD68 stain is positive showing numerous histiocytes and macrophages, which is typical in GC reactions. It is important to note the keratin and PAX8 stains were both negative demonstrating the absence of RCC recurrence as shown in Figures 3(c) and 3(d), respectively.

GC reactions have been noted to be enhanced on both contrast-enhanced CT and MRI thereby mimicking cancer recurrence [8] (Figure 2(a)). Various GC reactions mimicking recurrent or metastatic cancer have also been observed in bone and brain tissues as primary tumors and in colon tissue secondary to hemicolectomy [9–11]. For these reasons, patients can benefit from biopsy or fine needle aspiration prior to resection when suspicious lesions develop. The literature is void of substantial reports noting the frequency of GC reactions following ablation therapy of other organs.

## 4. Conclusion

Our report describes the delayed presentation of tumefactive fat necrosis and multinucleate GC reaction following cryoablation of clear cell RCC. This case demonstrates the importance of considering every possibility when presented with a patient that develops new perinephric abnormalities following ablation. This knowledge, combined with thorough evaluation, may aid in avoiding unnecessary invasive treatments for suspicious but nonmalignant lesions following ablation treatments.

## Abbreviations

CT: Computed tomography
GC: Giant cell
MRI: Magnetic resonance imaging
RCC: Renal cell carcinoma.
